# On Mons. Cadet's Unwholesomeness of Tea

**Published:** 1810-10

**Authors:** J. C. Mottles

**Affiliations:** London


### *For the Medical Journal.*

### *On Mons. Cadet's Unwholesomeness of Tea.*

HAVING paid some attention to the natural history, and medicinal qualities of Tea, I opened your last Journal with the hope of acquiring information from Mons. Cadet's account of its unwholesomeness.

As tea affords a beveridge I daily enjoy with pleasure, I was gratified in finding, from his chymical analysis, that instead of evincing its unwholesomeness, the title of his essay, it appears to prove its wholesomeness, more cogently than perhaps any other performance on the subject of this exotic. It proves, that it contains gallic acid and tansins, which may be considered as properties calculated to strengthen, rather than to debilitate the constitution. Who would say, that Peruvian bark is unwholesome because it contains tansius? or that wheat and potatoes are debilitating because they contain animal gluten?

Although the experiments of Mons. Cadet are favourable to the wholesomeness of tea, yet chemical analysis does not fully determine the dietetic qualities of substances. If we refer to three articles in general use, and consequently very universally known, salt-petre, common salt, and sulphur, what information as to their wholesomeness would chemical analysis convey? The public might be told, that they contain aquafortis, spirit of sea salt, and oil of vitriol; that a few drops of any of these taken inwardly would excoriate the throat and stomach; or, applied externally, would burn their clothes to rags, and even destroy the hardest stones; or combined, dissolve gold itself; yet, experience confirms the cook in the use of salt-petre, and common salt; and the apothecary of sulphur.

The most general objection against tea, is that of its sedative and debilitating power, which the experiments of Mons. Cadet, if they mean any thing, would tend to controvert. This supposed sedative power has been attempted to be established on experiments also, which I shall extract from an inaugural thesis, printed in 1769, and leave your medical readers to reconcile them with those of Mons. Cadet in 1809.

I.

"In abdominis cavitatem, atque membranam cellulosum, ranæ injeci circiter tres drachmas aquæ stillatitiæ odoratæ. Post viginti minuta alterum ranæ crus, seu pes posterior, multum adficiebatur, dum parum mobilitatis, aut sensibilitatis, monstrabat, quæ adfectio per quatuor horas perseverabat, et rana in statu torpido insensili universalis ultra novem horas manebat, donec gradatim ad pristinam vigorem rediret. Simili ratione liquorem a distillatione theæ viridis superstitem, atque ulteriori evaporatione magis concentratum injeci, sed inde nullum effectum sensibilem inductum vidi.

II.

" a Nerves Ischiaticis ranæ denudatis, atque cavitati abdominis, aquam stillatitiam fragrantem adplicui, intra dimidiam horam extremitates posteriores penitus paralyticæ insensilesque deveniebant, et post horæ circiter spatium ranæ vivere desiit.

b "Liquorem a distillatione residuum, eadem ratione alii ranæ admovi, sed nullos inde natos observare potui effectus sedantes, immo virtutem magis stimulantem, quam sedativam, præstare videbatur.

"Exc "Extractum in aqua solutum, et sub iisdem conditionibus, iisdem partibus admotum, nullum effectum sensibilem produxit."

J. C. MOTTLES.

*London, September* 5, 1810.

